# Preparation and Evaluation of Nanoemulsion of Citronella Essential Oil with Improved Antimicrobial and Anti-Cancer Properties

**DOI:** 10.3390/antibiotics12030478

**Published:** 2023-02-27

**Authors:** Talha Jawaid, Ali Mohammed Alaseem, Mohammed Moizuddin Khan, Beenish Mukhtar, Mehnaz Kamal, Razique Anwer, Saif Ahmed, Aftab Alam

**Affiliations:** 1 Department of Pharmacology, College of Medicine, Imam Mohammad Ibn Saud Islamic University (IMSIU), Riyadh 13317, Saudi Arabia; 2 Department of Basic Medical Science, College of Medicine, Dar Al Uloom University, Riyadh 13314, Saudi Arabia; 3 Department of Physiology, Santosh Deemed to be University, Ghaziabad 201009, India; 4 Department of Pharmaceutical Chemistry, College of Pharmacy, Prince Sattam Bin Abdulaziz University, Al-Kharj 11942, Saudi Arabia; 5 Department of Pathology, College of Medicine, Imam Mohammad Ibn Saud Islamic University (IMSIU), Riyadh 13317, Saudi Arabia; 6 Department of Physiology, College of Medicine, Imam Mohammad Ibn Saud Islamic University (IMSIU), Riyadh 13317, Saudi Arabia; 7 Department of Pharmacognosy, College of Pharmacy, Prince Sattam Bin Abdulaziz University, Al-Kharj 11942, Saudi Arabia

**Keywords:** anti-cancer, antimicrobial, citronella, essential oils, nanoemulsion

## Abstract

The development of new pharmaceutical solutions for treating various diseases results from a growing understanding of the benefits of using essential oils. One of the most often used volatile materials among essential oils is the oil of the citronella plant, termed citronella essential oil (CITEO), which has potential for use in food and medicine. Its wide use is limited due to lipophilicity, high volatility and poor physicochemical stability. With this background, the present study aims to evaluate the properties of CITEO-nanoemulsion (CITEO-NE) by analyzing its antimicrobial activities against *Staphylococcus aureus* (*S. aureus*) and *Candida albicans* (*C. albicans*) and its anticancer activity against, human skin adenocarcinoma cell line (A431). The CITEO-NE was prepared and evaluated for the size range of 130 ± 5 nm, polydispersity index (PDI) of 0.127 and zeta potential −12.6 mV. The percentage % of entrapment efficiency (%EE) of nanoemulsions loaded with CIT was very high at the beginning of the study, at 95.5 ± 4.775%. The MIC was observed to be 500 µg/mL for CITEO and 250 µg/mL for CITEO-NE against *S. aureus* and 250 µg/mL for CITEO and 125 µg/mL for CITEO-NE against *C. albicans*. The time-kill assay also suggests the effectiveness of CITEO-NE against the test pathogens as a novel alternative therapy. The IC_50_ values of CITEO and CITEO-NE exhibited significant cytotoxic properties against the A431 cell line, with 41.20 μg/mL and 37.71 μg/mL, respectively. Hence, our findings revealed that encapsulation of CITEO increased the pharmacological properties.

## 1. Introduction

Essential oils (EOs) are also known as ethereal oils or volatile oils [1]. They are derived from natural plants, including leaves, flowers, grains, and even flower buds, and belong to a distinct class of heterogeneous secondary metabolites [2]. Natural volatile extracts of plant substances known as EOs have great export potential due to their popularity in the food and pharmaceutical industries. In the Middle and Far East, plant essences and extracts, from which our current EOs have evolved, have been widely used in Rome, Greece, Egypt and other ancient civilizations for many centuries [3]. On the global market, there are at least 150 different kinds of essential oils that are marketed [2].

In modern aromatherapy, the essential oils of a variety of herbs and spices are used because these oils have been shown to possess a wide range of biological activities, especially the great potential to act as antimicrobial, antifungal, antiviral, analgesic, anticarcinogenic, antiparasitic, anti-inflammatory, and antioxidant agents [4]. For this reason, the essential oil chosen was that contained in the leaves of the test plant for this study, citronella, known scientifically as *Cymbopogon winterianus* Jowitt [5]. Citronella essential oil (CITEO) has been recommended for a variety of purposes, since the FDA, FEMA, and independent studies have all agreed that citronella oil and its main ingredient geraniol are safe [6]. Citronella oil and its constituents are used extensively in the manufacture of soaps, detergents, and fragrances; it is also used as a flavoring in foods [7]. It is useful in medicine as an analgesic, anticonvulsant, and anxiolytic, among other things, and it is also an agent for fighting off infections caused by fungi, bacteria, parasites, and worms [8].

Essential oils are chemical substances that are easily destroyed by light or temperature fluctuations. Consequently, encapsulating essential oils is a strategy used to prevent deterioration and decrease unwanted interactions with other components of the formulation. Encapsulation not only preserves the chemical properties and biological activity but also allows them to be released into the medium in a controlled manner. This prevents the rapid loss of substances that would otherwise occur due to the volatility of essential oils [8,9,10]. Although a significant amount of work has been published on the citronella plant, one of the plant’s limiting issues is its high volatility, which makes it difficult to release CITEO in a controlled way [11]. Recently, there has been a substantial amount of effort devoted to the development of nanoemulsions as a delivery mechanism for bioactive compounds derived from plants. Therefore, it has been proposed to use nanoscale CITEO emulsions to improve their industrial use by lowering the required doses [12]. Despite the growing literature on nanoemulsions, there are relatively few papers in which plant oils have been used; this is probably because these oils are insoluble in water. This restriction can be overcome by encapsulating these oils in water–oil emulsions or nanoemulsions [13]. 

In response to this, throughout the course of the last several decades, growing research efforts have concentrated on the investigation of nanoemulsions as well as the formulation processes involved in nano-emulsification [14,15,16]. A nanoemulsion is a colloidal dispersion of two immiscible liquids that is both isotropic and kinetically stable due to the presence of a surfactant. Its droplet size range of 20–200 nm indicates promising industrial applications and excellent stability for months [17]. The low complexity of these systems as well as their easy formulation procedures, potential for industrial scaling up, lack of toxicity, and promising biomedical or nanomedical applications are the primary reasons for this. Nanoemulsions are made of very tiny oil droplets that are maintained in aqueous environments by surfactants [18]. 

Agrawal et al. [11] successfully used cavitation-assisted techniques to synthesize nanoemulsion formulations of citronella. However, owing to their limited water solubility, the efficacy of CITEO nanoemulsion in antibacterial and anticancer applications has not yet been evaluated. Therefore, the aim of the present study was threefold: first, the development and evaluation of a CITEO-incorporated nano-sized emulsion suitable for application; second, comparative antimicrobial activities against *Staphylococcus aureus (S. aureus)* and *Candida albicans (C. albicans)*; third, the evaluation of anti-cancer activity against human skin adenocarcinoma cell line (A431). 

## 2. Results and Discussion

### 2.1. Morphological Study

**Figure 1:**
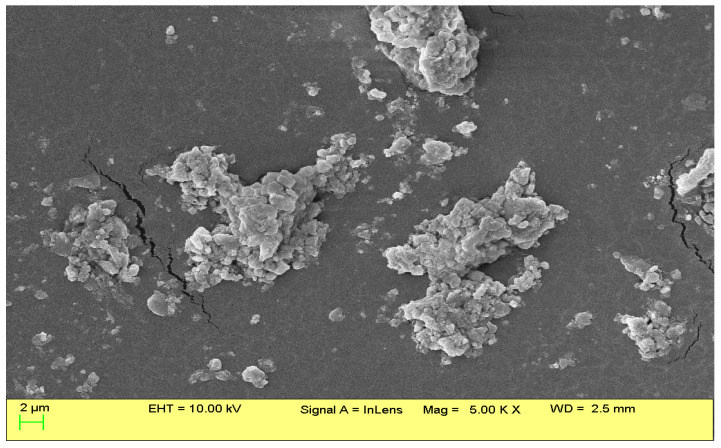
The SEM image of CITEO-loaded nanoemulsions.

The morphology and size of emulsion droplets are known to be affected by the emulsifier used, the type of oil, and the manufacturing method [19]. The SEM images obtained from the surface of the CITEO-loaded nanoemulsions in this study are depicted in Figure 1. The dried CITEO-loaded nanoemulsions had a non-uniform structure and aggregation among the particles. This shape is likely attributed to the drying process of the sample prior to SEM imaging. Aggregates of droplets were also observed, which might be caused by droplet flocculation or insufficient sublimation of water during sample preparation.

### 2.2. Size Distribution, Particle Size, and Zeta Potential

**Figure 2:**
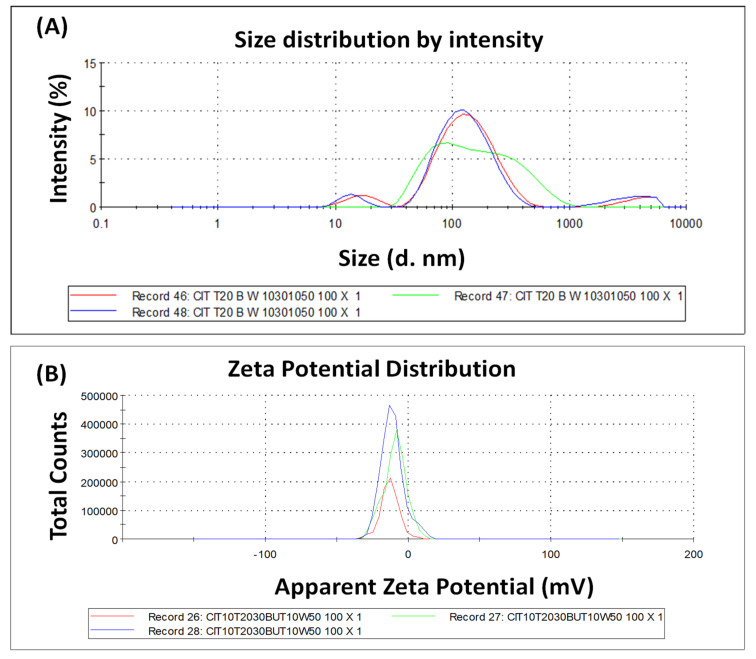
(**A**) Particle size and size distribution; (**B**) Zeta size of CITEO-loaded nanoemulsion.

The DLS technique was used to accomplish measurements of the size and polydispersity index of CITEO-loaded nanoemulsions; the results of these measurements are shown in Figure 2A. The mean hydrodynamic diameter of CITEO-loaded nanoemulsions were found to be less than 200 nm. The CITEO-loaded nanoemulsion displayed small oil droplet size, with the particle size of 130 ± 5 nm and a polydispersity index (PDI) of 0.127. The PdI indicates that bacterial samples are generally quite polydispersed, with a broad size distribution. The globule size of CITEO-loaded nanoemulsions observed in dynamic light scattering (DLS) studies are in agreement with what we observed in the scanning electron microscopic evaluations. The Zetasizer nano ZS (Malvern, UK) was used to experimentally determine the Zeta potential of loaded CITEO nanoemulsions by evaluating the electrophoretic mobility of the oil globules or the rate at which they move towards opposing electrodes when subjected to electric fields. As shown in Equation (1), a higher zeta potential means a higher electrophoretic mobility or speed.

(1) $$\mu e=\frac{2z\varepsilon f\left(ka\right)\;}{3\eta \;}$$

The zeta potential of CITEO-loaded nanoemulsions was found to be −12.6 mV, as shown in Figure 2B. The presence of a high surface charge is thought to be the cause of the nanoemulsions’ excellent stability at high zeta potential values, since particles with potentials greater than +30 mV and lower than 30 mV are regarded to be stable. This minimizes the likelihood of coagulation owing to electrostatic repulsion between particles with identical electric charges, prolongs the system’s stability, and facilitates re-dispersion [20].

### 2.3. Percentage Entrapment Efficiency (%EE)

The percentage EE of nanoemulsions loaded with CITEO was very high at the beginning of the study, at 95.5 ± 4.775%. After 30 days, we again determined the %EE of nanoemulsions loaded with CITEO and observed 94.1 ± 4.705%. This means that after 30 days of storage, no significant change (*p* > 0.05) in the %EE was observed. These results clearly show that the preparation of a nanoemulsion of CITEO helps to maintain EO despite its volatility.

### 2.4. Antimicrobial Activity

The comparative antibacterial assay of CITEO and CITEO-NE was performed in *S. aureus* bacteria using the well diffusion technique by taking different concentrations of 0.1%, 0.15%, 0.2%, and 0.25%, as shown in Table 1. The results clearly indicate that citronella nanoemulsion showed best zones of inhibition against *S. aureus,* with 19.3 ± 0.7 mm diameters at 0.2% concentration as compared to Citronella oil. Citronella oil showed the best zones of inhibition of against *S. aureus,* 12.5 ± 2.1 mm in diameter at 0.2% concentration. The MIC was observed to be 0.05% for CITEO and 0.025% for CITEO-NE against *S. aureus.* The case of a standard drug, i.e., amoxycillin, showed 34 mm of ZOI against *S. aureus*. After this, we determined the antifungal activity of the prepared formulation, i.e., CITEO-NE and bare CITEO, against *Candida albicans* (*C. albicans*) in concentrations of 0.1%, 0.15%, 0.2%, and 0.25%. The results clearly indicate that CITEO-NE showed the best anti-fungal activity against *C. albicans* with 25.6 ± 1.4 mm of ZOI, whereas in the case of bare CITEO, ZOI was observed to be 22 ± 1.4 mm at a concentration of 0.2%. The MIC was observed to be 0.025% for CITEO and 0.0125% for CITEO-NE against *C. albicans.*

**Figure 3:**
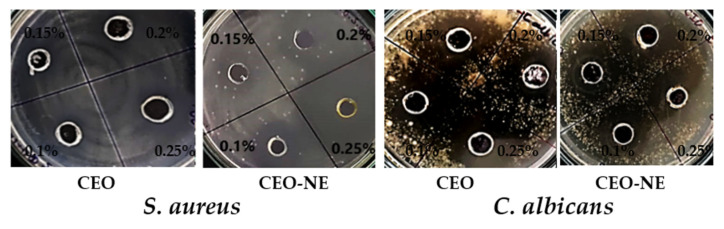
Antimicrobial activity of citronella essential oil (CITEO) and its nanoemulsion (CITEO-NE) against *S. aureus.* and *C. albicans*.

From the reports in Figure 3, it is clear that nanoemulsions loaded with CITEO exhibited a larger bacterial and fungal inhibitory zone compared to bare CITEO. The data (Table 1) also show that the formulations with the highest concentrations of nanoparticles had the widest growth-free halo, indicating the antibacterial capabilities of CITEO-NE. In addition, we found that a higher concentration of CITEO and CITEO-NE resulted in a larger zone of inhibition. These results indicate that CITEO-NE interacts with the bacterial cell. The results showed that CITEO-NE was more effective than CITEO against *S. aureus* and *C. albicans*.

### 2.5. Time-Kill Analysis

**Figure 4:**
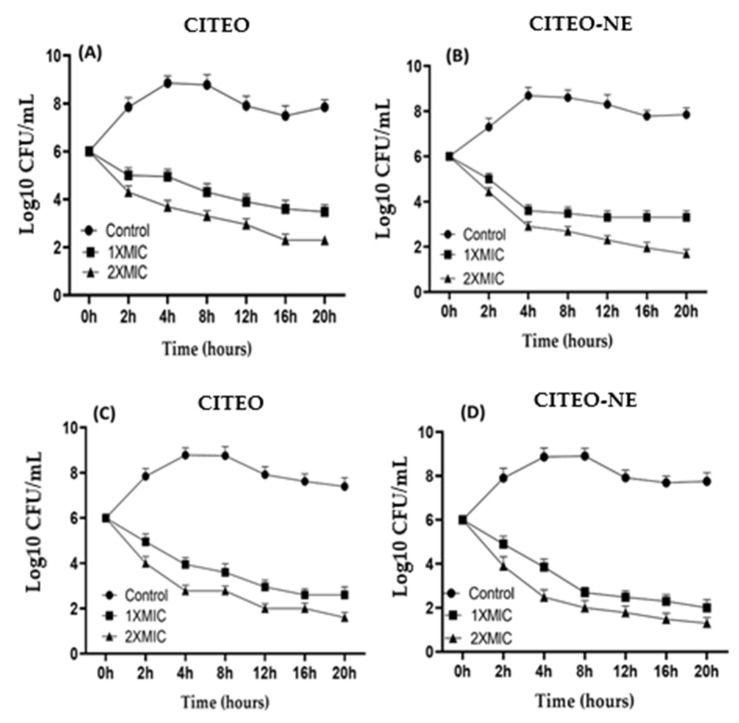
Time-killing assays of (**A**) CITEO against *S. aureus*, (**B**) CITEO-NE against *S. aureus*, (**C**) CITEO against *C. albicans* and (**D**) CITEO-NE against *C. albicans*.

The effect of CITEO and CITEO-NE on the growth curve of bacteria and fungi was evaluated separately in the culture medium of *S. aureus* and *C. albicans*. This was done to determine the effect of CITEO and CITEO-NE on the growth curve as shown in Figure 4. The findings for the control group, which received no therapy, were 6 to 8.85 log10 CFU/mL growth. In contrast, *S. aureus* growth was significantly reduced in the treatment given CITEO and CITEO-NE during the first 2 to 12 h. The results of treating *S. aureus* with CITEO remained the same at about 3.5 to 3.9 log10 CFU/mL and 2.3 to 2.9 log10 CFU/mL with 1× MIC (0.05%) and 2× MIC (0.1%), respectively, as shown in Figure 3A. The growth of *S. aureus* in the untreated group was 6 to 8.69 log10 CFU/mL after 4 h of treatment with CITEO-NE. The growth of *S. aureus* in the 1× MIC treatment group was 3.33 log10 CFU/mL, while in the 2× MIC treatment groups was, it was 1.69 to 2.3 log10 CFU/mL, as shown in Figure 3B. 

In a similar manner, we carried out the comparative, time-kill assay of CITEO and CITEO-NE against *C. albicans*. The time-kill assay was observed to be 6 to 8.8 log10 CFU/mL in the case of the vehicle treatment. After receiving CITEO, the development of *C. albicans* was significantly inhibited in the first 4 to 8 h after treatment. It remained the same at approximately 2.6 × log10 CFU/mL to 2.95 × log10 CFU/mL when treated with 1× MIC and 1.6 × log10 CFU/mL to 2 × log10 CFU/mL when treated with 2× MIC, as shown in Figure 3C. Treatment with CITEO-NE against *C. albicans,* remained constant at approximately 2 × log10 CFU/mL to 2.5 × log10 CFU/mL and 1.3 × log10 CFU/mL to 1.78 × log10 CFU/mL for 1× MIC and 2× MIC treatment, respectively. The results showed that CITEO-NE is more effective than pure CITEO, as shown in Figure 3D. Hence, the summary of the time-kill assay suggests that the effectiveness of CITEO-NE against the test pathogens as a novel alternative therapy.

### 2.6. Anticancer Activity

**Figure 5:**
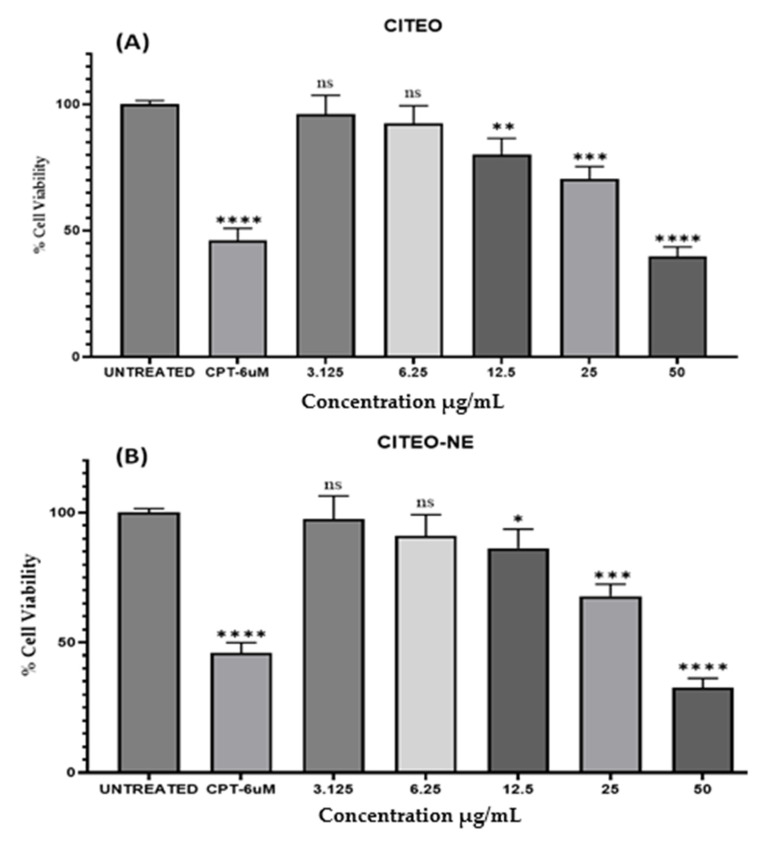
Cytotoxicity assay of CITEO (**A**) and CITEO-NE (**B**) with different concentrations against A431. *p*-value (* *p* < 0.05, ** *p* < 0.01, *** *p* < 0.001 and **** *p* < 0.0001; ns: not significant) when compared to the untreated group using two-way ANOVA of GraphPad prism 9.5.0.

To investigate the anticancer effect of CITEO and CITEO-NE in vitro, cell viability and cytotoxicity were tested in A431 (human skin adenocarcinoma cell line), as shown in Figure 5. The statistical data of cell cytotoxicity by MTT indicate that the test compounds, i.e., CITEO and CITEO-NE, exhibited significant cytotoxic properties against the A431 cell line, with IC_50_ values of 41.20 ± 3.8 µg/mL and 37.71 ± 4.2 µg/mL, respectively. Camptothecin (IC_50_ value of 28.6 ± 3.2 µg/mL) was used as the standard for the study. Among the two test compounds, CITEO-NE was considered to be effective against skin cancer due to its lower IC_50_ value. The results clearly show that the CITEO-NE–based drug delivery system can be delivered to targeted tumor cells with higher efficiency compared to CITEO. 

## 3. Materials and Methods

Citronella essential oil (CITEO) was procured from Indian essential oil (New Delhi, India), while nutrient media and Sabouraud dextrose agar media was purchased from HiMedia (Mumbai, India). Tween 80, MTT reagent, Dimethyl sulfoxide (DMSO), and Camptothecin (CPT) were purchased from Sigma Merck (St Louis, MO, USA). The other reagents or chemicals used in the study were analytically graded. *Staphylococcus aureus* (ATCC 25923) and *Candida albicans* (ATCC 10231) were collected from the Department of Pharmaceutics, College of Pharmacy, Prince Sattam Bin Abdulaziz University (PSAU), Al-Kharj. Solvents and other chemicals were also purchased from Sigma Merck. The human skin adenocarcinoma cell line A431 was obtained as gift from Dr. Mohammad Raish, Department of Pharmaceutics, King Saud University (Riyadh, Saudi Arabia).

### 3.1. Nanoemulsion Preparation of Essential Oils

We prepared the CITEO-loaded nanoemulsions (CITEO-NE) using a probe sonicator (VCX 750, SONICS, Wallingford, CT, USA). For this, we took CITEO as an oil phase, Tween 20 as an emulsifier, and Butanaol as a co-emulsifier. We took distilled water as an aqueous phase in different proportions: 10%, 30%, 10%, and 50% *v/v*. We then left the combination in a cold bath. It was then sonicated with a 6 mm probe at an amplitude of 60% for 3 min with on and off cycles of 10 s. Nanoemulsions were kept at room temperature to make sure they were stable and there was no formation of phase separation or creaming.

### 3.2. Study of Physical Appearance and Morphology

For the characterization of morphology, we carried out scanning electron microscopy (SEM; Hitachi High Technologies, Hillsboro, OR, USA). To observe the morphology of the outer surface of CITEO-NE, we kept the samples on a polycarbonate substrate. They were left at room temperature to dry and then were kept in a critical dryer. After drying, the samples were coated with gold and further examined under SEM [21].

### 3.3. Size Distribution, Particle Size, and Zeta Potential

To observe the particle size and size distribution, the dynamic light diffusion (DLS) instrument (Malvern Instruments, Malvern Worcestershire, UK) technique was utilized in conjunction with the Zetasizer Nano ZS, model ZEN3500. To analyze the sample, we took 10 µL of the CITEO-NE and 990 microliters of deionized water. These were mixed properly to create a dilution of 100 times. After this, the sample was placed in a cuvette and further analyzed at 25 °C. We then determined the zeta potential of the CITEO-NE using a Zetasizer Nano ZS. Zeta sizing was carried out by the use of a one-of-a-kind mixed-mode measurement or phase analysis light scattering technique. This measurement enables high-precision determination for both the average zeta potential and the distribution (M3-PALS) [22].

### 3.4. Percentage Entrapment Efficiency (%EE)

To determine the %EE of CITEO in CITEO-NE, the desired concentration of the mixture was ensured by combining 100 mg of CITEO with 10 mL of NE solution and then agitating the mixture at 1000 rpm for 60 min. After this, to load the CITEO into the nanoemulsion, we kept the prepared solution mixture at room temperature for 24 h. After 24 h, the mixture was centrifuged at 5000 rpm for 30 min. After this, the supernatant solution and CITEO-NE were separated. The recovered sample was further dispersed in Milli-Q water before using [23]. Using a UV-vis spectrophotometer (Shimadzu, Kyoto, Japan) at the λmax value at 360 nm, the concentration of the CITEO supernatator was calculated. The following formula was used to calculate the entrapment effectiveness of CITEO:

(2) $$\%\;\mathrm{EE}=\frac{\left(\mathrm{Total}\;\mathrm{amount}\;\mathrm{of}\;\mathrm{CITEO}\;\mathrm{added}-\mathrm{Amount}\;\mathrm{of}\;\mathrm{free}\;\mathrm{CITEO}\;\mathrm{in}\;\mathrm{supernatent}\right)}{\mathrm{Total}\;\mathrm{amount}\;\mathrm{of}\;\mathrm{CITEO}\;\mathrm{added}}\;\times 100$$

### 3.5. Determination of Minimum Inhibition Concentration (MIC) and Minimum Bactericidal Concentration (MBC) 

#### 3.5.1. Preparation of Nutrient Media

To prepare the nutrient media, we used 1 liter of distilled water to dissolve 28 g of the nutrient media. Before the sterilization process began, the pH of the media was measured. After, this the nutrient media was sterilized in an autoclave at 121 ℃ for 15 min. After completion of the sterilization process, the nutrient media was cooled. After cooling, the nutrient media was poured into the plates. It was then placed in the laminated air flow until the agar became film.

#### 3.5.2. Antibacterial Activity

We determined out the antibacterial activity of the CITEO and CITEO-NE in bacteria *S. aureus* by the well diffusion technique. In this, we first prepared 10 mg of the standard stock solution of amoxicillin in 100 mL of distilled water to obtain 0.01% of the standard solution. After that, 25 mg of test samples of CITEO and CITEO-NE were prepared in 10 mL of solvent (5% dimethyl sulfoxide (DMSO)) to obtain a 0.25% test solution. After this, the inoculum was prepared; the test organism *S. aureus* was inoculated with 10 mL of nutrient broth. Bacterial suspensions were standardized at 10^8^ CFU/mL bacteria and were kept in shakers. Afterward, 50 µL of the broth inoculum (10^8^ CFU/mL) was removed with a micropipette and transferred to fresh sterile solidified agar media plates [24]. On a sterilized agar plate, the inoculation was uniformly spread using a sterile spatula. Using a clean cork cleaner, four holes measuring 6 millimeters were drilled into the medium that was to be inoculated. Samples with different concentrations, from 0.1% to 0.25%, were placed in a well plate. On a separate plate, 50 µL of the reference drug was added. The samples were then incubated at 37 °C for 18–24 h. It was then allowed to diffuse at room temperature for 30 min. Following this, we incubated the sample and observed the formation of a discrete zone around the wells corresponding to the antibacterial activity of the antibiotic tested. We observed the zone of inhibition (ZOI) in millimeters (mm) according to the method described in [25].

#### 3.5.3. In Vitro Antifungal Activity

##### Preparation of Sabouraud Dextrose Agar (SDA) Media

Sabouraud dextrose agar media (65.0 g) was dissolved in 1 L of distilled water to prepare the SDA medium. Before sterilization of the prepared media, we measured the pH. After measuring the pH, media was autoclaved at 121 °C at 15 lb pressure for 15 min for sterilization. After completion of sterilization, media was kept for cooling without solidification. The SDA medium was then poured onto the plates and then placed in a laminar air flow until the agar became firm. This procedure was repeated until the agar was completely set.

##### Antifungal Activity

We tested the antifungal activity of the prepared formulation (CITEO and CITEO-NE sample) in *C. albicans.* For this purpose, a culture of *C. albicans* was first spread on the prepared SDA medium. To prepare a standard stock solution of fluconazole with a concentration of 0.01% and the test solution with a concentration of 0.1% to 0.25%, CITEO and CITEO-NE were dissolved in 5% DMSO. After this, we followed all the steps discussed in this manuscript in Section 3.5.2 using the method given by Mohammadi-Sichani et al., 2012 [24]. 

### 3.6. Minimum Inhibitory Concentration (MIC) 

The MIC of prepared formulations (CITEO and CITEO-NE) was evaluated using the agar well diffusion test [26]. In this technique, an inoculum of 50 μL of selected bacteria *S. aureus* and fungus *C. albicans* was seeded onto sterile Mueller Hinton (MH) agar plates. Then, a well of 6 mm in diameter was drilled into the agar with the use of a sterile cork borer. The well was filled with 50 μL of different concentrations of CITEO and CITEO-NE samples prepared in 5% DMSO. After depositing the different sample concentrations into the wells, they were kept at room temperature for 30 min and then at 37 °C for 24 h. Measurements were taken in mm to determine the circular inhibition zones. The MIC was calculated by microdilution, and the test was performed in MH broth. We prepared different dilutions of the test samples; 100 μL aliquot was diluted in MH broth containing 10^6^ CFU/mL. Different plates were incubated at different temperatures, i.e., the plates of *S. aureus* containing bacteria were kept at 30 °C ± 5 °C/24 h and 25 °C ± 5 °C for *C. albicans.* After 24 h, the lowest CITEO and CITEO-NE concentration that completely inhibited microbial growth was considered the MIC. 

### 3.7. Time-Kill Analysis

Time-kill kinetics of the chosen formulations (CITEO and CITEO-NE) were determined by adapting a method from a published paper [27]. We took two different concentrations equivalent to 1× MIC (0.05% and 0.025%) and 2× MIC (0.1% and 0.05%) for the kill kinetic assay against *S. aureus*. Similarly, two different concentrations equivalent to 1× MIC (0.025% and 0.0125%) and 2× MIC (0.05% and 0.025%) were selected for the kill kinetic assay against *C. albicans*. An inoculum of size 1 × 10^6^ CFU/mL was added to it and further incubated at 37 °C. At intervals of 0, 2, 4, 8, 12, 16, and 20 h, a total of 1 mm of inoculum was taken from the medium. The colony-forming units (CFUs) of bacterial cells were determined. A negative control with organisms and DMSO (without essential oils) was also evaluated. Assays were performed in triplicate, and time-kill plots were generated by calculating log10 CFU/mL of mean colony count versus time.

### 3.8. Anti-Cancer Activity

We carried out the anti-cancer activity by MTT assay against the Human Skin adenocarcinoma cell line (A431) using the test sample CITEO and CITEO-NE in the concentrations ranging from 3.125 µg/mL to 50 µg/mL. For this purpose, 200 µL of cell suspension was added to a 96-well plate at the required cell density (20,000 cells per well) without the assay medium. The cells allowed to grow for approximately 24 h. After this, the selected concentration of the test agents was added for incubation at 37 °C for 24 h in 5% CO_2_. The plates were removed from the incubator after completion of the incubation period. MTT reagents were added to a final concentration of 0.5 mg/mL of total volume after the spent medium was removed. In order to prevent the plates from being exposed to light, aluminum foil was used to cover them. After three hours in the incubator, the plates were removed. After removing the MTT reagents, 100 µL of a solution containing DMSO was added. Dissolution may be improved by using a gyro shaker to provide a gentle agitation. The absorption is measured using a spectrophotometer or an ELISA reader with a wavelength of 570 nm. The percent cell viability is calculated using the following formula:

% cell viability = Abs of treated cells/Abs of Untreated cells × 100(3)

The IC_50_ value was determined using a linear regression equation, i.e., Y = Mx + C, where Y = 50, and M and C values are derived from the viability graph.

## 4. Conclusions

Essential oils derived from plants could provide a natural, more effective alternative to antibiotics made from synthetic chemicals. Due to the exceptional pharmacological effects, citronella essential oil (CITEO) has attracted the most interest among these numerous compounds. Essential oils such as CITEO can be challenging to use in crop protection due to their poor water solubility, high volatility, and instability. These are the three main physicochemical properties that contribute to this difficulty. Nanoformulation provides an answer to a number of the major problems associated with EOs. These include the low water solubility of EOs, their volatility, and their chemical instability. By preparing and analyzing nanoemulsion of CITEO, the current study aimed to achieve its primary goal of finding solutions to the previously identified problems. According to the data described above, encapsulation of CITEO in nanoemulsions is an effective method to achieve this goal. Compared with uncoated CITEO, encapsulation of CITEO in nanoemulsion is one of the most promising strategies to enhance pharmacological activities such as antibacterial and anticancer effects. On the other hand, further studies are needed to evaluate the potential impact of CITEO-based nanoformulations for different diseases.

## Figures and Tables

**Table 1:** Comparative zone of inhibition (means ± SD) and minimum inhibitory concentration (MIC) study of CITEO and CITEO-NE against *S. aureus* and *C. albicans*.

Concentration (%)	CITEO		CITEO-NE	
	*S. aureus*	*C. albicans*	*S. aureus*	*C. albicans*
**0.1**	5 ± 0.8	8.3 ± 2.1	13 ± 0.7	10.6 ± 0.7
**0.15**	7.5 ± 0.2	11.6 ± 1.4	15 ± 0.7	14 ± 1.4
**0.2**	10 ± 1.4	15.6 ± 1.4	16.3 ± 0.7	20.3 ± 1.4
**0.25**	12.5 ± 2.1	22 ± 1.4	19.3 ± 0.7	25.6 ± 1.4
**MIC**	500 µg/mL	250 µg/mL	250 µg/mL	125 µg/mL

## Data Availability

The data presented in this study are available on request from the corresponding author.
